# Case Report: Effect of low energy availability and training load on sleep in a male combat sport athlete

**DOI:** 10.3389/fspor.2022.981755

**Published:** 2023-01-06

**Authors:** Craig Thomas, Carl Langan-Evans, Mark Germaine, Mario Artukovic, Helen Jones, Craig Whitworth-Turner, Graeme L. Close, Julien Louis

**Affiliations:** ^1^ Research Institute for Sport and Exercise Sciences (RISES), Liverpool John Moores University, Liverpool, United Kingdom; ^2^ School of Sport, Exercise and Health Sciences (SSEHS), Loughborough University, Loughborough, United Kingdom; ^3^ SFI Centre for Research Training in Machine Learning, Dublin City University, Dublin, Ireland; ^4^ Department of Athletics, University of Pennsylvania, Philadelphia, PA, United States

**Keywords:** weight making, energy restriction, energy expenditure, training load, actigraphy

## Abstract

**Purpose:**

The aim of this case report was to describe the sleep responses in a male combat sport athlete, who was engaging in both chronic (CWL) and acute (AWL) weight loss practices in order to reduce body mass for a national competition.

**Methods:**

During the first seven weeks of training (Phases 1 and 2), the athlete adhered to a daily energy intake (EI) equating to their resting metabolic rate (1700 kcal·day^−1^) followed by a reduction in EI (915–300 kcal·day^−1^) in the 5 days before weighing in (Phase 3). Nocturnal sleep was monitored throughout the 8-week training period using wristwatch actigraphy and frequent measurements of body mass/composition, daily exercise energy expenditure and training load (TL) were taken.

**Results:**

The athlete was in a state of low energy availability (LEA) during the entire training period. There was a very large decrease in LEA status during phase 3 compared with phases 1 and 2 (3 vs. 20 kcal·kgFFM·day^−1^) and there was a small decrease in TL during phase 3 compared with phase 2 (410 vs. 523 AU). The athlete's sleep efficiency increased throughout the training period, but total sleep time displayed a small to moderate decrease in phase 3 compared with phases 1 and 2 (386 vs. 429 and 430 min). However, correlational analysis demonstrated trivial to small, non-significant relationships between sleep characteristics and the athlete's LEA status and TL.

**Conclusion:**

These findings suggest that CWL and AWL practices that cause fluctuations in LEA and TL may be implemented without compromising the sleep of combat sport athletes.

## 1. Introduction

Professional and amateur combat sport disciplines are organised into weight categories to promote fair competition between opponents of equal body mass (BM) (1). Despite this, combat sport athletes often aim to compete in the lowest weight category possible *via* a process known as *weight making*, in an attempt to gain competitive advantages over opponents of smaller proportion (2). To that end, there is a widespread culture of reducing BM within combat sports, with athletes across a range of disciplines typically losing 4–10 kg leading up to competition (3). These losses are achieved by engaging in both acute (AWL—over hours or days) and chronic (CWL—over several weeks or months) weight loss practices, which include a combination of increasing exercise energy expenditure (EEE), restricting energy intake (EI) and techniques to induce specific states of dehydration (4). The AWL approach, when not well considered, has been associated with a number of health risks, as a rapid reduction in BM may affect fluid balance, thermoregulation, and metabolism, as well as cause injury or in extreme cases even death (4). When the goal is to reduce BM gradually, combat sport athletes may utilise a CWL approach by inducing an energy deficit resulting in losses of 0.5–1 kg·week^−1^ (1, 5, 6). However, a reduction in EI with concomitant increases in EEE *via* escalations in overall daily training load (TL), is likely to result in a state of low energy availability (LEA), which has been associated with a number of psycho-physiological consequences as characterised within the Athlete Triad (TRIAD) and Relative Energy Deficiency in Sport (RED-S) models (7–9).

Sleep is a key factor in health and wellbeing, which influences a number of psycho-physiological systems (10). Varying sleep parameters have been correlated with a range of performance markers when assessed in an athletic combat sport population (11). To maintain optimal health and daytime functioning, it is recommended adults achieve at least 420 min of total sleep time with ≥85% sleep efficiency per night (12, 13). As previously described, an increase in daily TL is an inherent consequence of adopting a CWL approach. Proliferated training periods may result in greater sleep duration, sleep efficiency and slow wave sleep percentage (14, 15), whilst others have demonstrated either no change or reductions in sleep duration and efficiency (16–18). It has been suggested that sleep may improve with subtle changes in TL but become disturbed with large increases relative to the athlete (19). Whilst the TL of combat sport athletes may not be as high compared to athletes from other sports (20), due to BM elevations induced partly from inactivity (5, 21), these athletes are often exposed to a large increase in TL when making weight. This is supported by data in combat sport athletes, where sleep was rated worse during early preparation training compared with mid preparation training and on training days compared with rest days, indicating sleep may be challenged when commencing training after a rest period (22, 23). Thus, it is plausible that an inflation in TL associated with weight making may negatively impact sleep and subsequently the training and competition readiness of combat sport athletes, concomitant with LEA.

Despite the potential impact of increased TL and LEA, sleep is not considered within the aforementioned TRIAD and RED-S models, yet this may be of concern given a lower sleep duration may affect appetite, mood, and performance (24–26). Indeed, in male and female dancers who were screened for LEA using the Dance Specific Energy Availability Questionnaire, among those with sleep problems, 37% reported difficulty falling asleep and 21% indicated disrupted sleep patterns (27). Previously, when compared to high level athletes from non-weight making sports (e.g., racquet sports), a greater number of combat sport athletes perceived themselves to have had a sleep problem at some point in their lifetime (28). Furthermore, AWL practices may induce sleep disturbances in combat sport athletes leading into competition, yet current evidence has suggested this may not be the case when utilising techniques such as low fibre/residue diets and water loading across time periods of up to 5 days (29). Such findings suggest that sport specific factors are responsible for sleep differences between sporting demographics and in the case of combat sport athletes, associated periods of LEA may potentially result in sleep disturbances.

Therefore, the aim of this case report was to describe the sleep responses in a male combat sport athlete, who was engaging in both CWL and AWL practices in order to make weight for a national competition. During an 8-week training period the athlete's sleeping patterns were monitored using wristwatch actigraphy and frequent measurements of BM/composition, daily EEE and TL were assessed, in tandem with controlled restriction of daily EI. This analysis was part of a larger case report, which evaluated the effects of LEA on health and performance related indices associated with the Male TRIAD and RED-S models (5).

## 2. Materials and methods

### 2.1. Athlete overview

The athlete (age: 19 years; stature: 166 cm; BM: 72.5 kg) was a Caucasian male taekwondo competitor with 5 years international competition experience, who would habitually use CWL and AWL practices over time periods of 3–4 weeks to lose 4–5 kg, so they could compete in the <68 kg feather weight category. For this case report, the athlete aimed to compete in the <63 kg bantam weight category at the British University Championships, therefore requiring a reduction of >9.5 kg BM across an 8-week training period (5). Prior to commencement of the training period, the athlete did not use any sleep aids (e.g., medication) or work night shifts, nor had they travelled across different time zones. Additionally, throughout the training period they worked part-time, did not partake in any daytime napping, disclosed that they understood the importance of sleep for optimising performance and wanted to gain an insight into their sleeping patterns. The athlete provided written informed consent and institutional ethical approval was granted by Liverpool John Moores University.

### 2.2. Athlete assessment

#### 2.2.1. Measures of energy availability status, daily training load and TRIAD/RED-S consequences

BM was measured each morning, post void of the bladder/bowels and determined to the nearest 0.01 kg on a calibrated digital scale, with measures of stature established to the nearest 0.1 cm using a free-standing stadiometer (Seca 702 & 123; Seca GmbH, Hamburg, Germany). To examine energy availability (EA) status, measures of body composition inclusive of bone mineral content/density, absolute and relative fat mass, and fat free mass (FFM) were assessed using Dual-energy x-ray Absorptiometry (QDR Series Discovery A, software version 12:4:3, Hologic Inc, Bedford, MA, USA), as per best practice guidelines (30). Gross EEE from all training sessions was measured *via* a combined heart rate and actigraphy unit (Actiheart 4, CamNtech Ltd, Cambridgeshire, United Kingdom) and calculated into net EEE as previously described (5). EI was provided for the athlete throughout the entire 8-week training period as highlighted in the following sections. Daily EA was subsequently calculated using the equation: EI minus net EEE divided by FFM with values <30 kcal·kgFFM·day^−1^ classified as LEA (31). Daily TL was assessed using the session rating of perceived exertion (s-RPE) method, based on Borg's Category Ratio 10 scale (1–10 arbitrary units) (32). Further details pertaining to the measurement of TRIAD/RED-S health and performance consequences inclusive of resting metabolic rate, cardiac function, maximal dynamic strength and power, cardiorespiratory capacity, psychological state, and blood clinical chemistry (for biomarkers related to endocrine, bone turnover, hydration, electrolyte, renal, liver, and lipid profiles) are highlighted in Langan-Evans et al. (5).

#### 2.2.2. Wristwatch actigraphy

To assess nocturnal sleep throughout the 8-week training period, the athlete was provided with a wrist activity monitor (Actiwatch 4, Cambridge Neurotechnology Ltd, Cambridgeshire, United Kingdom) for the first four weeks and then again for the last four weeks, which was set to an epoch length of 1 min at a medium sensitivity. Actigraphy has shown to have a good overall agreement with polysomnography (90%–91%; gold standard measurement of sleep) and is a valid alternative for sleep/wake estimation (33). On each night, the athlete was instructed to wear the activity monitor on their non-dominant wrist 30–60 min before they retired to bed in their home environment. Upon their bedtime, they recorded the clock time in the Core Consensus Sleep Diary (CSD) (34) and pressed the marker button on the activity monitor. The following morning, they pressed the marker button again upon their final awakening and filled in the remainder of the Core CSD. During the monitoring period, the athlete received no sleep hygiene education. The recorded markers from the activity monitor and the information from the Core CSD were then used to determine bedtime, sleep onset, final awakening time and get up time, so that sleep behaviour could be automatically calculated using the appropriate software (Actiwatch Activity and Sleep Analysis version 5.24, Cambridge Neurotechnology Ltd, Cambridgeshire, United Kingdom). From the analysis, the following sleep characteristics were used: bedtime (BT—h:min), final awakening (FA—h:min), time in bed (TIB—min), sleep onset latency (SOL—minutes between bedtime and sleep onset), total sleep time (TST—min), wake after sleep onset (WASO—min) and sleep efficiency (SE—total sleep time as a percentage of time in bed (sleep quality)).

### 2.3. Dietary and training periodisation

#### 2.3.1. Phase 1 (−8 week to −4 week) & phase 2 (−4 week to −1 week)

**Figure 1:**
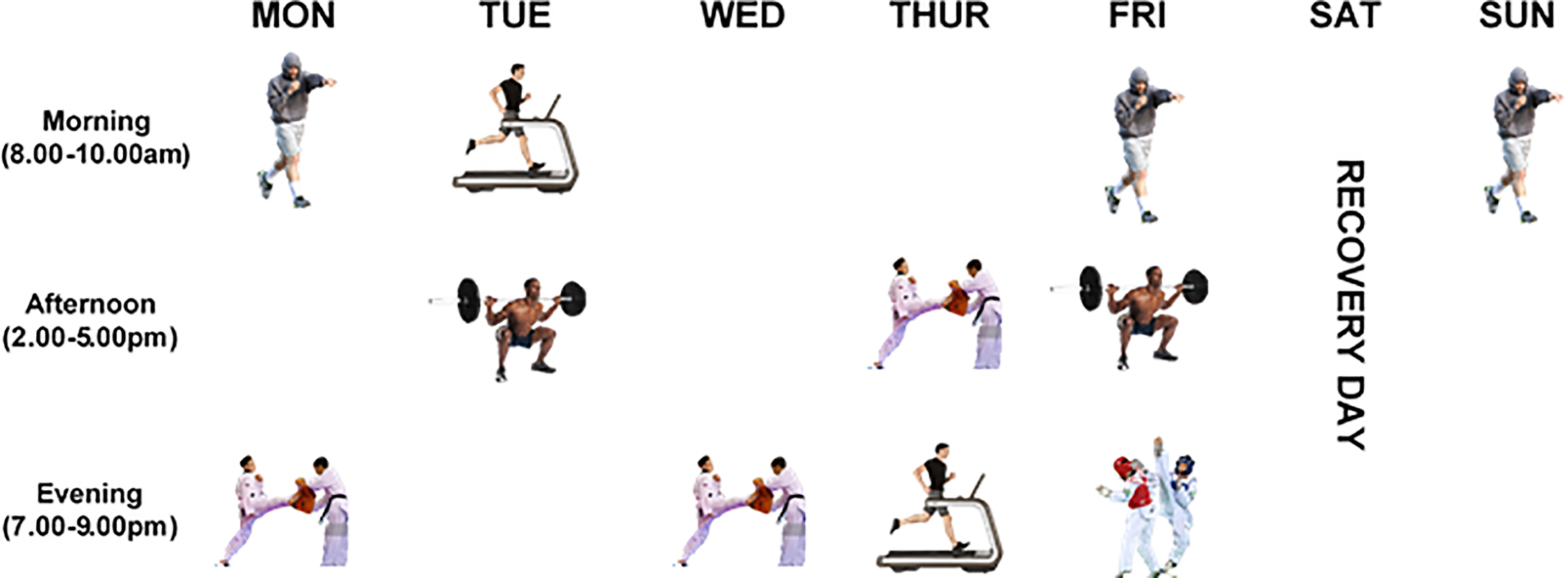
Weekly training schedule during phase 1 (−8 WK to −4 WK) & phase 2 (−4 WK to −1 WK). Training consisted on average of 3 × 60 min aerobic running (Monday, Friday & Sunday), 2 × 30 min high intensity interval treadmill running (Tuesday & Thursday), 2 × 60 min strength and conditioning (Tuesday & Friday), 3 × 120 min taekwondo specific technical/tactical (Monday, Wednesday & Thursday) and 1 × 120 min sparring (Friday) based sessions.

During the first seven weeks of the training period, the athlete ingested a daily EI that equated to their resting metabolic rate measurement at baseline (1700 kcal·day^−1^) (See Table 1), consisting of pre-packaged meals totalling 3.4 g·kgFFM^−1^ carbohydrate (CHO) (748 kcal·day^−1^), 2.3 g·kgFFM^−1^ protein (506 kcal·day^−1^) and 0.9 g·kgFFM^−1^ fat (446 kcal·day^−1^) (5). Alongside restriction in EI, the athlete completed 12–15 h·week^−1^ of set periodised training across a weekly schedule, consisting of three aerobic, two high intensity interval, two resistance and three taekwondo specific sessions with one sparring-based session. All strength and conditioning sessions were prescribed and delivered by an accredited strength and conditioning coach, with over 10 years' experience. Sport specific sessions were prescribed and delivered by an Olympic licensed coach with over 12 years' experience. During Saturdays no training occurred, and these were classified as recovery periods [see Figure 1 for schedule and Langan-Evans et al. (5) for further detail on exercise prescription].

**Table 1:** Measurements of energy availability, intake, expenditure, training load and sleep throughout the training period.

Time phase/ characteristic	Monday	Tuesday	Wednesday	Thursday	Friday	Saturday	Sunday	Combined Average	Effect Size
***PHASE 1 (−8 WK TO −4 WK)***									
*EA (kcal·kgFFM·day^−1^)*	15 ± 1	20 ± 1	24 ± 0	20 ± 1	9 ± 1	31 ± 0	24 ± 0	**20 ** **±** ** 7**	**t: VL**
*EI (kcal·day^−1^)*	1700 ± 0	1700 ± 0	1700 ± 0	1700 ± 0	1700 ± 0	1700 ± 0	1700 ± 0	**1700 ** **±** ** 0**	**t: VL**
*EEE (kcal·day^−1^)*	873 ± 47	584 ± 31	409 ± 22	617 ± 34	1209 ± 66	0 ± 0	376 ± 20	**559 ** **±** ** 370**	**t: T**
*TL s-RPE (AU)*	728 ± 388	481 ± 212	439 ± 229	304 ± 250	1140 ± 268	0 ± 0	191 ± 21	**469 ** **±** ** 375**	**t: T**
*BT (h: min)*	23:53 ± 00:17	23:45 ± 00:11	23:25 ± 00:35	23:43 ± 00:12	00:02 ± 00:17	23:34 ± 00:51	23:52 ± 00:07	**23:44 ** **±** ** 00:12**	
*FA (h: min)*	07:22 ± 00:54	06:23 ± 00:42	06:56 ± 01:45	07:23 ± 00:44	08:23 ± 01:27	08:47 ± 02:49	07:54 ± 01:35	**07:35 ** **±** ** 00:49**	
*TIB (min)*	458 ± 56	404 ± 36	472 ± 151	465 ± 38	517 ± 88	558 ± 131	507 ± 128	**483 ** **±** ** 49**	**s: M**
*TST (min)*	417 ± 30	361 ± 52	418 ± 97	406 ± 44	465 ± 74	490 ± 155	448 ± 92	**429 ** **±** ** 42**	**t: M**
*SE (%)*	91.6 ± 5.1	89.1 ± 6.7	90.1 ± 8.7	87.1 ± 3.1	90.3 ± 5.3	86.4 ± 10.2	89.0 ± 6.9	**89.1 ** **±** ** 1.8**	**l: VL**
*WASO (min)*	26 ± 22	28 ± 20	20 ± 16	46 ± 7	23 ± 24	42 ± 34	29 ± 21	**31 ** **±** ** 10**	**l: VL**
*SOL (min)*	6 ± 2	9 ± 4	13 ± 11	9 ± 9	13 ± 4	22 ± 14	5 ± 4	**11 ** **±** ** 6**	**t: M**
***PHASE 2 (−4 WK TO −1 WK)***									
*EA (kcal·kgFFM·day^−1^)*	13 ± 1	22 ± 1	25 ± 0	20 ± 1	7 ± 1	31 ± 0	23 ± 0	**20 ** **±** ** 8**	**VL**
*EI (kcal·day^−1^)*	1700 ± 0	1700 ± 0	1700 ± 0	1700 ± 0	1700 ± 0	1700 ± 0	1700 ± 0	**1700 ** **±** ** 0**	**VL**
*EEE (kcal·day^−1^)*	968 ± 52	527 ± 29	340 ± 18	616 ± 34	1333 ± 72	0 ± 0	430 ± 23	**579 ** **±** ** 418**	**T**
*TL s-RPE (AU)*	710 ± 615	507 ± 78	357 ± 6	600 ± 137	1270 ± 139	0 ± 0	220 ± 9	**523 ** **±** ** 407**	**S**
*BT (h: min)*	23:38 ± 00:31	23:47 ± 00:06	23:50 ± 00:09	23:57 ± 00:07	00:01 ± 00:06	00:30 ± 00:30	00:10 ± 00:17	**23:59 ** **±** ** 00:17**	
*FA (h: min)*	05:40 ± 00:04	06:16 ± 01:00	05:42 ± 00:15	08:11 ± 00:14	08:18 ± 01:45	10:29 ± 01:06	08:09 ± 01:11	**07:32 ** **±** ** 01:45**	
*TIB (min)*	372 ± 32	399 ± 74	362 ± 16	508 ± 23	510 ± 108	629 ± 97	481 ± 57	**466 ** **±** ** 95**	**S**
*TST (min)*	340 ± 27	373 ± 64	342 ± 12	462 ± 11	473 ± 105	558 ± 90	459 ± 61	**430 ** **±** ** 81**	**S**
*SE (%)*	91.4 ± 2.3	93.7 ± 3.5	94.5 ± 1.6	91.0 ± 3.8	92.6 ± 2.1	88.8 ± 5.6	95.3 ± 3.2	**92.5 ** **±** ** 2.3**	**M**
*WASO (min)*	13 ± 4	5 ± 6	5 ± 8	20 ± 2	16 ± 6	23 ± 9	7 ± 6	**13 ** **±** ** 7**	**S**
*SOL (min)*	10 ± 5	10 ± 7	5 ± 1	12 ± 7	8 ± 4	17 ± 16	13 ± 9	**11 ** **±** ** 4**	**M**
***PHASE 3 (5 DAYS PRIOR TO OCWI)***									
* *	−5 D	−4 D	−3 D	−2 D	−1 D	OCWI	COMP		* *
*EA (kcal·kgFFM·day^−1^)*	−4	9	8	7	−3	–	–	**3 ** **±** ** 6**	
*EI (kcal·day^−1^)*	915	915	863	863	300	–	–	**771 ** **±** ** 265**	
*EEE (kcal·day^−1^)*	1120	409	421	467	474	–	–	**578 ** **±** ** 304**	
*TL s-RPE (AU)*	1220	180	160	160	330	–	–	**410 ** **±** ** 458**	
*BT (h: min)*	00:04	00:04	23:56	23:51	00:09	–	–	**00:00 ** **±** ** 00:07**	
*FA (h: min)*	05:40	06:40	05:36	06:44	08:54	–	–	**06:42 ** **±** ** 01:20**	
*TIB (min)*	340	401	342	434	540	–	–	**411 ** **±** ** 82**	
*TST (min)*	313	373	328	408	507	–	–	**386 ** **±** ** 77**	
*SE (%)*	92.1	93.0	95.9	94.0	93.9	–	–	**93.8 ** **±** ** 1.4**	
*WASO (min)*	13	11	6	1	13	–	–	**9 ** **±** ** 5**	
*SOL (min)*	10	12	6	4	5	–	–	**7 ** **±** ** 3**	

EA, energy availability; EI, energy intake; EEE, exercise energy expenditure; TL s-RPE, training load session rating of perceived exertion; BT, bedtime; FA, final awakening; TIB, time in bed; TST, total sleep time; SE, sleep efficiency; WASO, wake after sleep onset; SOL, sleep onset latency; WK, week; D, day; OCWI, official competition weigh in; COMP, competition day.

t, trivial; s, small; l, large effect size compared to Phase 2 (−4 WK to −1 WK); T, trivial; S, small; M, moderate; VL, very large effect size compared to Phase 3 (5 days prior to OCWI).

#### 2.3.2. Phase 3 (5 days prior to weigh in)

In the 5 days prior to the weigh in daily EI was gradually reduced (see Table 1), whereby the athlete consumed 0.5 g·kgFFM^−1^ CHO (110 kcal·day^−1^), 1.8 g·kgFFM^−1^ protein (385 kcal·day^−1^) and 0.9 g·kgFFM^−1^ fat (420 kcal·day^−1^) on days −5 and −4, followed by 0.3 g·kgFFM^−1^ CHO (67 kcal·day^−1^), 1.8 g·kgFFM^−1^ protein (400 kcal·day^−1^) and 0.8 g·kgFFM^−1^ fat (396 kcal·day^−1^) on days −3 and −2 (5). On −1 day, the athlete had one meal in the morning, which contained 1.4 g·kgFFM^−1^ protein (300 kcal·day^−1^). This reduction in EI corresponded with a taper to training volume, therefore clamping EEE (see Table 1). From 12:00 h on −1 day until 13:00 h at the official competition weigh in the following day, the athlete then abstained from all food and fluids, and participated in self-directed AWL methods as further described in Langan-Evans et al. (5).

### 2.4. Statistical analysis

All phase 1 and 2 data highlighted in Table 1 are presented as mean ± SD and phase 3 are individual values. To compare between phases, quantifications of daily EA, EI, EEE and TL s-RPE alongside sleep measures of TIB, TST, SE, WASO and SOL were assessed *via* Hedges *g* effect size calculations utilising the following quantitative criteria to explain the practical significance of the findings: trivial <0.2, small 0.2–0.59, moderate 0.6–1.19, large 1.20–1.99, and very large ≥2.0 (35). Given the non-normal distribution of data, sleep measurement variables of TIB, TST, SE, WASO and SOL were also examined in comparison to both daily EA and TL s-RPE *via* a non-parametric Spearman's rank correlation coefficient employing the following criteria to explain the relationship of association: trivial <0.1, small 0.1–0.29, moderate 0.3–0.49, large 0.5–0.69, very large 0.7–0.89 and almost perfect 0.9–1.00 (35). All analyses were performed using Prism version 9.2.0 (GraphPad, San Diego, California, USA) and the alpha level was set at *p* < 0.05.

## 3. Results

As highlighted in Table 1, the athlete was classified as being in a state of LEA during the entire training period, with no difference (0 kcal·kgFFM·day^−1^; ES = 0.00) during CWL across phases 1 and 2. LEA was further augmented during AWL within phase 3, resulting in very large differences (−17 kcal·kgFFM·day^−1^) compared to both phase 1 (ES = 2.37) and 2 (ES = 2.16). This pattern of LEA was dictated predominantly by changes in EI, which remained unchanged between phases 1 and 2 (0 kcal·day^−1^; ES = 0.00) and was followed by a very large reduction (−929 kcal·day^−1^; ES = 5.12) during phase 3. To that end, modulations in LEA status were independent of changes in EEE, with minimal variances between 20 to −1 kcal·day^−1^ across all three phases, resulting in trivial differences (ES ≤ 0.05). Finally, measures of TL s-RPE also mirrored EEE, with trivial differences between phases 1 and 2 (54 AU; ES = 0.12), 1 and 3 (−59 AU; ES = 0.13) and a small difference between phases 2 and 3 (−113 AU; ES = 0.24).

Also highlighted in Table 1, the athlete's TIB gradually reduced across the training period, resulting in a small difference between phases 1 and 2 (−17 min; ES = 0.20), a small difference between phases 2 and 3 (−55 min; ES = 0.56) and a moderate difference between phases 1 and 3 (−72 min; ES = 1.03). Independent of reductions in TIB, TST between phases 1 and 2 remained similar resulting in a trivial effect (1 min; ES = 0.01). However, these differences begin to increase by both small and moderate effects when comparing reductions in TST between phases 2 and 3 (−44 min; ES = 0.51) and 1 and 3 (−43 min; ES = 0.68), respectively. In contrast to TIB and TST, there is a subsequent improvement in SE through each phase, with a large difference between phases 1 and 2 (3.4%; ES = 1.43), very large difference between phases 1 and 3 (4.7%; ES = 2.63) and a moderate difference between phases 2 and 3 (1.3%; ES = 0.60). Measures of WASO also follow a similar pattern to improvements in SE, with continuing reductive improvements of a large effect between phases 1 and 2 (−18 min; ES = 1.81), a very large effect (−22 min; ES = 2.43) between phases 1 and 3 and a small effect (−4 min; ES = 0.59) between phases 2 and 3. Finally, SOL remained stable between phases 1 and 2 (0 min; ES = 0.00), with a decreasing moderate difference between phases 1 and 3 (−4 min; ES = 0.74) and a moderate difference between phases 2 and 3 (−4 min; ES = 1.02), respectively.

**Figure 2:**
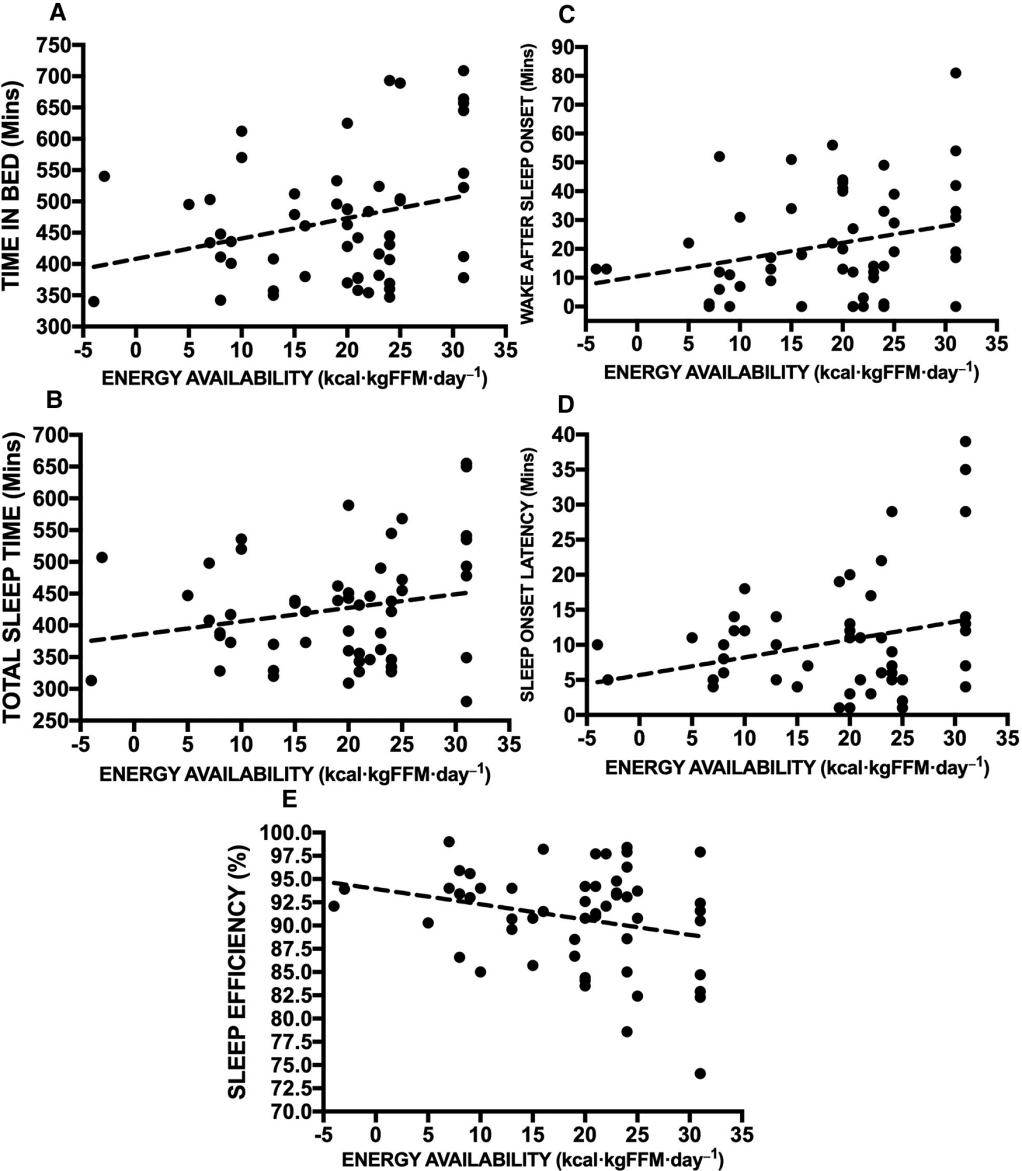
Rank correlation coefficient assessments between daily energy availability status and sleep measures inclusive of TIB (**A**), TST (**B**), WASO (**C**), SOL (**D**) and SE (**E**).

**Figure 3:**
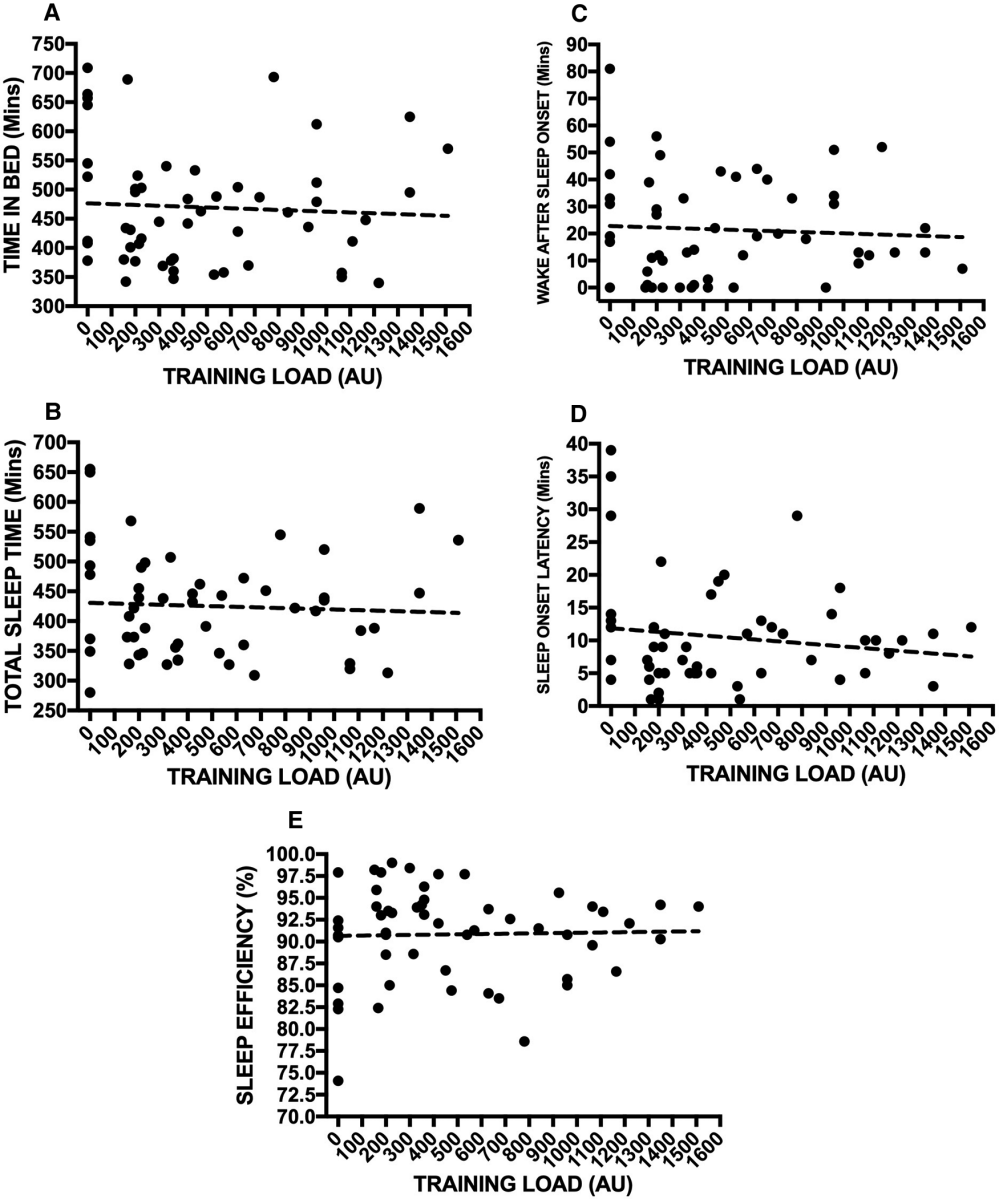
Rank correlation coefficient assessments between s-RPE daily training load and sleep measures inclusive of TIB (**A**), TST (**B**), WASO (**C**), SOL (**D**) and SE (**E**).

Figure 2 highlights the association between the differing sleep measures and daily EA status, demonstrating small, non-significant, positive relationships with TIB (2A. r 0.23; *p* = 0.10), TST (2B. r 0.19; *p* = 0.18), WASO (2C. r 0.21; *p *= 0.13), SOL (2D. r 0.12; *p* = 0.40) and a negative relationship with SE (2E. r −0.19; *p* = 0.16). Finally, Figure 3 also showcases the same analysis with a focus on sleep measures and daily TL, yet again highlighting trivial to small, non-significant, negative relationships with TIB (3A. r −0.11; *p* = 0.44), TST (3B. r −0.10; *p* = 0.46), WASO (3C. r −0.01; *p* = 0.94), SOL (3D. r −0.08; *p* = 0.58) and a positive relationship with SE (3E. r 0.01; *p* = 0.92).

## 4. Discussion

The aim of this case report was to describe the sleep responses in a male combat sport athlete, who was engaging in both CWL and AWL practices in order to make weight for a national competition. During the first seven weeks of training, the athlete's TST and SE were, on average, in line with current sleep recommendations (12, 13). When comparing between the first two phases, TST was similar, but the athlete did display an increase in SE during phase 2, which was a result of a small reduction in TIB coupled with a large reduction in WASO. The TST and SE values from phases 1 and 2 are comparable to non-athletes [431 min and 88.7%; Leeder et al. (36)] and greater than that of other individual sport athletes monitored during a typical out of competition training phase [390 min and 85.9%; Lastella et al. (37)]. In phase 3, where the athlete participated in AWL practices, a further reduction in TIB led to a moderate to very large increase in SE compared with the first two phases, however, TST decreased meaning the athlete fell short of the recommended 420 min per night (12). The TST achieved by the athlete in phase 3 is similar to other combat sport athletes who utilised low fibre/residue diets and water loading across 5-days (386 vs. 373 min); Dunican et al. (29), whilst the SE of the athlete was remarkably higher than those same combat sport athletes (93.8 vs. 79%); Dunican et al. (29). Our data suggests that combat sport athletes may achieve adequate sleep quality during the process of making weight, but that their sleep duration may be suboptimal in the days prior to competition due to changes in daily schedule.

This is the first report to assess nocturnal sleep in relation to daily EA and TL in a combat sport athlete using an objective sleep measurement. As expected, during the first seven weeks of the training period, the athlete was in a state of LEA (20 kcal·kgFFM·day^−1^). During the AWL in phase 3, there was a further reduction in EA (3 kcal·kgFFM·day^−1^) compared with the first two phases, due to a very large reduction in daily EI rather than changes in EEE. Despite this, correlational analysis demonstrated small, non-significant, relationships between the athlete's LEA status and sleep characteristics, suggesting that LEA may not be strongly related to the sleep of the athlete in this case report. This opposes the idea that disrupted nocturnal sleep may be used as an indicator of LEA status (27). With there being an inherent increase in TL from a CWL approach, this case report also assessed whether TL may impact sleep, independent of LEA. There was a small reduction in daily TL during phase 3 compared with phase 2, however in a similar manner to LEA, there were trivial to small, non-significant, relationships between this metric and the sleep characteristics of the athlete. This concurs with Caia et al. (16) where a higher daily TL did not result in changes to sleep but contrasts with Hausswirth et al. (17) in which sleep was disturbed in overreached triathletes that undertook longer training sessions. To that end it could be postulated that the athlete's TL in this case report was not large enough to impact their sleep (19). Collectively, our findings indicate that practitioners may implement CWL and AWL practices that induce fluctuations in LEA and TL without compromising the sleep of combat sport athletes.

Our case report also highlights that the athlete altered their sleep schedule during the AWL phase, which likely explains the reduction in TST in the days before competition. In comparison to the first two phases, there was a small to moderate reduction in TIB during phase 3 because of an earlier FA time. Anecdotally, the athlete stated that they had taken on more work-based commitments (due to less scheduled training sessions) that required an earlier time of FA. This is in broad agreement with studies that have shown that early training start times may constrain the time of FA and subsequently impact sleep duration (38, 39). In the case of the athlete in this report, the replacement of training with more work hours can be attributed to them not being at the highest level in their sport, nonetheless, this highlights the need for practitioners to be aware of an athlete's schedule outside of training. Given the importance of adequate sleep for physiological and psychological restoration (10), it is unknown whether the reduction in TST in the days before competition affected the athlete's competitive readiness. Although it should be noted that the athlete won a total of 4 contests to secure a gold medal placing at the championships. Therefore, more research is warranted to understand the individual sleep need of combat sport athletes and the minimum amount of sleep loss that may result in performance decrements.

## 5. Conclusions & practical implications

In conclusion, sleep duration decreased whilst sleep quality increased across the 8-week training period, though neither daily EA nor TL strongly correlated with overall sleep characteristics, suggesting practitioners may utilise CWL and AWL strategies without compromising sleep. TST fell below sleep guidelines during phase 3 and it is likely this was a product of an earlier FA imposed by the athlete's work schedule; thus, we recommend practitioners are aware of their athlete's schedule outside of training during the competition week in order to optimise the athlete's readiness for competition. The strength of this case report is that daily EI was controlled, and an objective measure of sleep was used, but there are some limitations. Our results reflect only one individual combat sport athlete, and it is possible that LEA and/or TL may impact sleep in other athletes. Nocturnal sleep was monitored across an 8-week training period, however, as sleep was not monitored prior to this, it is unknown whether the athlete's sleep had been further disrupted in comparison to their habitual sleeping patterns. Furthermore, it would have been beneficial to have monitored sleep at certain time points using polysomnography, given this is the gold standard measurement for sleep. Future research should examine the effect of daily EA and TL on sleep in larger combat sport athlete cohorts and across subsequent weight making cycles.

## Data Availability

The raw data supporting the conclusions of this article will be made available by the authors, without undue reservation.
